# The Modulating Effect of Dietary Beta-Glucan Supplementation on Expression of Immune Response Genes of Broilers during a Coccidiosis Challenge

**DOI:** 10.3390/ani11010159

**Published:** 2021-01-12

**Authors:** Islam I. Omara, Chasity M. Pender, Mallory B. White, Rami A. Dalloul

**Affiliations:** 1 Avian Immunobiology Laboratory, Department of Animal and Poultry Sciences, Virginia Tech, Blacksburg, VA 24061, USA; islam.omara@agr.cu.edu.eg (I.I.O.); ccox1@vt.edu (C.M.P.); mallw11@vt.edu (M.B.W.); 2 Animal and Poultry Division, Department of Animal Production, Faculty of Agriculture, Cairo University, Giza 12613, Egypt; 3 Department of Poultry Science, University of Georgia, Athens, GA 30602, USA

**Keywords:** β-glucan, broiler, cytokine, coccidiosis, immunity

## Abstract

**Simple Summary:**

Avian coccidiosis is the leading parasitic disease in the poultry industry and means to control its damages continue to be explored. This study evaluated the feeding effects of a yeast-derived β-glucan on expression of immune response genes in the spleen, thymus, and bursa of commercial broiler chickens during an *Eimeria* challenge. The study consisted of two dietary treatments (0% or 0.1% β-glucan) each with or without a coccidiosis challenge. There were significant effects from dietary β-glucan, *Eimeria* challenge, and their interaction for several gene targets in the spleen, thymus, and bursa on days 10 and 14 of age. Based on the current results, supplementation of dietary β-glucan in *Eimeria*-challenged birds enhanced and modulated the expression of immune response genes during coccidiosis.

**Abstract:**

This study investigated the effects of a yeast-derived β-glucan (Auxoferm YGT) supplementation on mRNA expression of immune response genes in the spleen, thymus, and bursa of broiler chickens during a mixed *Eimeria* infection. Day (d)-old chicks (*n* = 1440) were fed diets containing 0% or 0.1% YGT. On d 8 post-hatch, half the replicate pens (*n* = 8) were challenged with a mixed inoculum of *E. acervulina*, *E. maxima*, and *E. tenella*. On d 10 and d 14 post-hatch, the spleen, thymus, and bursa were collected to evaluate mRNA abundance by quantitative real-time PCR. Data were analyzed using PROC GLIMMIX model (2-way interaction) and differences were established by LS-MEANS with significance reported at *p* ≤ 0.05. In spleen tissues at d 10, expression of interleukin (IL)-10 and inducible nitric oxide synthase (iNOS) were elevated in both 0.1% YGT-fed challenged and non-challenged birds. In thymus tissues at d 14, expression of IL-10, IL-17F, interferon (IFN)-γ, iNOS, and macrophage migration inhibitory factor (MIF) were elevated in challenged birds fed 0.1% YGT. In bursal tissues at d 10 and d 14, expression of IL-10, IFN-γ, iNOS (d 10 only), and MIF were elevated in 0.1% YGT-fed challenged and non-challenged birds. Dietary β-glucan supplementation to chicken diets modulated their immune response to the *Eimeria* challenge.

## 1. Introduction

Poultry coccidiosis is considered the leading parasitic disease in commercial production with significant economic impact on the industry worldwide that is estimated at a devastating $3.2 billion annually [1,2,3]. Coccidiosis is an infection of the small intestine caused by intracellular protozoan parasites of the genus *Eimeria* often resulting in localized lesions leading to reduced nutrient utilization and performance in livestock and poultry [4,5]. Current control measures include primarily anticoccidial drugs and live oocyst vaccines; but, more natural feed supplements are being widely investigated as potential means of alleviating the impact of coccidiosis in poultry [6,7]. One of such additives is β-glucans, which are glucose polymers and can be derived from fungi, yeast, and cell walls of bacteria, as well as from cereal grains, including oat and barley [4]. Large variations exist in the structure of β-glucans from these different sources that ultimately result in differences in their physiological functions [8]. β-glucans from fungal and yeast sources are most effective in enhancing protective immunity against infectious agents due to their highly branched structure [9,10], as they have shown the ability to minimize coccidial infection in both growing pullets and layer chickens [11]. β-glucans from dried algae can help improve gut immunity and host protection, reducing lesion scores in broilers challenged with three prevalent species of *Eimeria* (*E. acervulina*, *E. maxima*, and *E. tenella*) [12].

β-glucans can have beneficial effects on both the innate and adaptive immune responses. Exposure to β-glucans has demonstrated enhanced proliferation and phagocytizing efficiency of leukocytes, including macrophages [13,14,15] and heterophils [16]. In terms of the adaptive immune response, β-glucans can enhance plasma levels of IgG and IgA, which indicates a heightened humoral response [17]. In addition, yeast β-glucans enhanced humoral and cell-mediated immune responses in broilers [4,18,19]. An increase in the size of primary and secondary lymphoid organs has also been reported due to dietary β-glucan supplementation, an additional indication of their immunomodulating capabilities [13,17]. Dietary β-glucans are also capable immunoprotective agents enhancing host defenses against coccidiosis [4,12,20], and protecting against other economically important bacterial (e.g., *Salmonella enterica*, *Escherichia coli*) and several viral pathogens (such as NDV, avian infectious bronchitis virus, and IBDV) [19,21,22]. Further, supplementation of yeast β-glucans improved gut health and enhanced host disease resistance by inducing endogenous gene expression of antimicrobial peptides in chickens challenged with necrotic enteritis [23].

Yeast-based β-glucans may be used as growth promoters and potential antibiotic alternatives in poultry against certain enteric pathogens by increasing populations of cells expressing secretory IgA, as well as goblet cells [24]. They can also modulate expression profiles of cytokines during a coccidiosis infection via enhanced innate and T helper Type I (Th1)-mediated immune response [4]. Furthermore, macrophages extracted from birds fed β-glucans exhibited higher expression levels of interleukin (IL)-1 [13], IL-2, interferon (IFN)-γ [17], IL-4, and IL-18 [25]. These results indicate the potential modulatory effect of dietary β-glucans via differential gene expression and warrant further research to evaluate their influence on the function of immune organs in chickens.

The objective of this study was to determine the effects of dietary supplementation of a β-glucan derived from the yeast *Saccharomyces cerevisiae* on the mRNA abundance of immune response genes in the major immune organs of broiler chickens with or without a coccidiosis challenge.

## 2. Materials and Methods

### 2.1. Birds, Diets, and Eimeria Challenge

On day (d) of hatch, 1440 straight-run Cobb 500 broiler chicks (non-vaccinated) were picked up from a commercial hatchery and transported to the Virginia Tech Turkey Research Farm. Chicks were placed into 48 floor pens (16 replicate pens/treatment) consisting of concrete floors and pine shavings with 30 chicks per pen (0.1 m^2^/bird). Chicks had ad libitum access to water and a non-medicated corn/soy-based starter diet in mash form containing 0 or 0.1% YGT Auxoferm (β-glucan extracted from *S. cerevisiae*, AB Vista, Marlborough, UK). The corn-soybean basal diet was formulated to meet or exceed the National Research Council (NRC) nutrient requirements for broilers [26]. On d 8 post-hatch, one half of the replicate pens (n = 8) was orally gavaged with 1 mL of a mixed inoculum containing 50,000 *E. acervulina*, 10,000 *E. maxima*, and 2500 *E. tenella* sporulated oocysts.

### 2.2. Tissue Sampling for Gene Expression Analysis

Eight male birds per treatment (1 bird/replicate) were sampled on d 10 and d 14 post-hatch (days 2 and 6 post-challenge). Sampled chicks were euthanized by cervical dislocation and their immune organs (spleen, thymus, and bursa) were aseptically excised, rinsed in ice-cold PBS, minced, snap-frozen in liquid nitrogen, and stored at −80°C until analysis.

### 2.3. Total RNA Extraction and Reverse Transcription

Total RNA was extracted from individual tissue samples with Trizol reagent and a Direct-zol™ RNA MiniPrep kit (ZYMO Research Inc., Irvine, CA, USA) according to the manufacturer’s instructions. First, tissue samples were removed from −80 °C and placed on dry ice. Then, a 20–30 mg aliquot of each sample was weighed, placed into a 2 mL microcentrifuge tube, and kept on ice until homogenization according to manufacturer’s recommendation. Following extraction, RNA was eluted by rinsing the column membrane twice with 25 μL of RNase-free water. Total RNA concentration was determined at optical density (OD) 260 (NanoDrop-1000, Thermo Fisher Scientific, Waltham, MA, USA), and RNA purity was verified by evaluating the ratio of OD 260 to OD 280. Total RNA was diluted to 0.2 μg/μL in nuclease-free water. Reverse transcription was accomplished using the high capacity cDNA Reverse Transcription kit (Applied Biosystems, Carlsbad, CA, USA) following the manufacturer’s protocol [27], and the cDNA was stored at −20 °C.

### 2.4. Quantitative Real-Time PCR

Quantitative real-time PCR (qRT-PCR) was performed using an ABI 7500 FAST Real-Time PCR System (Applied Biosystems). The cDNA was diluted 1:20 in nuclease-free water, and 1 μL of the diluted cDNA was added to each well of a 96-well plate. Next, 9 μL of PCR master mix containing 5 μL of Fast SYBR Green Master Mix (Applied Biosystems), 0.5 μL each of 2 μM forward and reverse primers, and 3 μL of sterile nuclease-free water per reaction were added to each well for a final volume of 10 μL. During the PCR reaction, samples were subjected to an initial denaturation phase at 95 °C for 20 s followed by 40 cycles of denaturation at 95 °C for 3 s and annealing and extension at 60 °C for 30 s. Gene expression for interleukin (IL)-10, IL-18, IL-17F, interferon (IFN)-γ, inducible nitric oxide synthase (iNOS), and macrophage migration inhibitory factor (MIF) was analyzed using glyceraldehyde-3-phosphate dehydrogenase (GAPDH) as an endogenous control. Each reaction was run in duplicate. Primers were designed (Table 1) using the Primer Express 3.0 software (Applied Biosystems) and synthesized by MWG Operon (Huntsville, AL, USA). qRT-PCR data were analyzed using the 7500 Real-Time PCR software (Applied Biosystems). Average gene expression relative to the GAPDH endogenous control for each sample was calculated using the 2^−ΔΔCt^ method [28]. The calibrator for each gene was the average ∆Ct value of the corresponding non-challenged, 0.0% YGT control group at d 10 and d 14 of each immune organ.

### 2.5. Statistical Analysis

Gene expression data were analyzed using the PROC GLIMMIX procedure of SAS (SAS Institute Inc., Cary, NC, USA). Statistical analysis was performed using the following PROC GLIMMIX model: main effects of dose (inclusion rate) and challenge, and their two-way interactions (dose × challenge) on d 10 and d 14. Following ANOVA, differences among experimental treatments were separated using a Tukey’s test and values were considered statistically different at *p* ≤ 0.05. The results are reported as least squares (LS) means ± standard error of the mean (SEM).

## 3. Results

### 3.1. IL-10 mRNA Expression Levels (Table 2; Figure 1)

**Figure 1:**
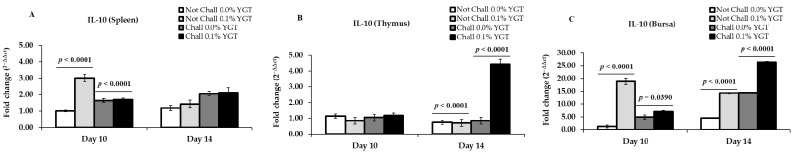
Effect of 2-way interaction (dietary β-glucan × *Eimeria* challenge) on abundance of IL-10 mRNA in the spleen (**A**), thymus (**B**), and bursa (**C**) of broiler chicks at d 10 and d 14. Relative gene expression (2^−∆∆Ct^) was calculated using the ∆∆Ct method with glyceraldehyde-3-phosphate dehydrogenase (GAPDH) as the endogenous control and the average ∆Ct value of unchallenged, 0.0% YGT control group at d 10 and 14 for each immune organ as the calibrator. Data are presented as least squares means + SEM. Not Chall = not challenged; Chall = challenged with *Eimeria*; YGT = Auxoferm YGT, *Saccharomyces cerevisiae*-derived β-glucan.

There was a significant interaction between the dietary β-glucan treatment and the *Eimeria* challenge on IL-10 mRNA levels in the spleen and bursa at d 10 and in the thymus on d 14 (Table 2; Figure 1A–C). In spleen (Figure 1A) and bursal (Figure 1C) tissues at d 10, expression was elevated by 3- and 19-fold (*p* < 0.0001, and *p* < 0.0001, respectively) in non-challenged birds fed the 0.1% YGT diet when compared with 0.0% YGT control birds. In challenged birds fed the 0.1% YGT diet, expression of IL-10 mRNA was elevated by 2- and 7-fold (*p* < 0.0001 and *p* = 0.0390, respectively) in spleen and bursal tissues when compared with 0.0% YGT controls. On d 14, there was a significant interaction on expression in the thymus, with a 6-fold increase observed in the challenged birds fed 0.1% YGT (*p* < 0.0001; Figure 1B). There was no significant interaction between diet and challenge on IL-10 mRNA expression in the thymus at d 10 or in the spleen and bursa by d 14.

**Table 2:** Main effects of dietary β-glucan treatment, *Eimeria* challenge, and their interactions on target genes at day 10 and day 14 (days 2 and 6 post coccidiosis challenge).

Gene Target ^1^	Organ	*p*-Value ^3^ at d 10			*p*-Value ^3^ at d 14		
		Diet ^2^	Challenge	Diet × Challenge	Diet ^2^	Challenge	Diet × Challenge
IL-10	Spleen	<0.0001	0.0345	<0.0001	0.5437	0.0020	0.6702
	Thymus	0.6790	0.4993	0.2482	<0.0001	<0.0001	<0.0001
	Bursa	<0.0001	0.0005	<0.0001	0.0014	0.0012	0.7347
IL-18	Spleen	0.2546	0.0055	0.0207	0.3002	0.0707	0.4103
	Thymus	0.0585	0.3737	0.4735	0.1183	0.9320	0.0763
	Bursa	0.2281	0.0114	0.5750	0.3330	0.7018	0.1474
IL-17F	Spleen	0.9937	0.0013	<0.0001	0.2069	0.6390	0.3660
	Thymus	0.6774	0.4761	0.3153	<0.0001	<0.0001	<0.0001
	Bursa	0.2800	0.0008	0.9596	0.0735	0.0048	0.3903
IFN-γ	Spleen	0.2719	0.0033	0.0792	0.1884	0.1676	0.0011
	Thymus	0.0774	0.0785	0.3234	<0.0001	<0.0001	<0.0001
	Bursa	<0.0001	<0.0001	0.1031	0.0192	0.0016	0.0152
iNOS	Spleen	0.0007	0.1142	0.0040	0.0466	0.0266	0.0635
	Thymus	0.8977	0.9987	0.6537	0.0037	0.0003	<0.0001
	Bursa	0.0009	0.0003	0.0095	0.2475	0.8261	0.4419
MIF	Spleen	0.8068	0.9164	0.0058	0.5617	0.9049	0.0100
	Thymus	0.0855	0.0139	0.3614	0.0408	0.0011	0.0274
	Bursa	0.0007	0.9470	0.0001	<0.0001	<0.0001	0.0022

^1^ IL = interleukin; IFN = interferon; iNOS = inducible nitric oxide synthase; MIF = macrophage migration inhibitory factor. ^2^ YGT = Auxoferm YGT (β-glucan extracted from *Saccharomyces cerevisiae*). ^3^ Significance noted at *p* ≤ 0.05.

When looking at the main effects of either diet or challenge, there were some significant differences. The main effect of including YGT in the diet was to increase IL-10 mRNA expression in the bursa compared to control-fed birds on d 14 (*p* = 0.0014; Figure 1C). The effect of challenge also increased expression in spleen and bursal tissues of challenged birds on d 14 (*p* = 0.0020 and *p* = 0.0012; Figure 1A,C).

### 3.2. IL-18 mRNA Expression Levels (Table 2; Figure 2)

**Figure 2:**
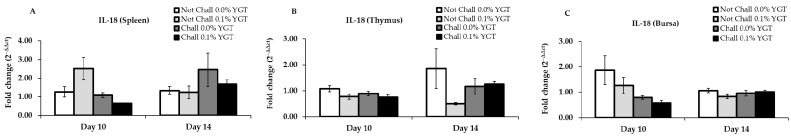
Effect of 2-way interaction (dietary β-glucan × *Eimeria* challenge) on abundance of IL-18 mRNA in the spleen (**A**), thymus (**B**), and bursa (**C**) of broiler chicks at d 10 and d 14. Relative gene expression (2^−∆∆Ct^) was calculated using the ∆∆Ct method with glyceraldehyde-3-phosphate dehydrogenase (GAPDH) as the endogenous control and the average ∆Ct value of unchallenged, 0.0% YGT control group at d 10 and 14 for each immune organ as the calibrator. Data are presented as least squares means + SEM. Not Chall = not challenged; Chall = challenged with *Eimeria*; YGT = Auxoferm YGT, *Saccharomyces cerevisiae*-derived β-glucan.

On d 10, there was a significant interaction between diet and challenge on IL-18 expression in the spleen (*p* = 0.0207; Table 2; Figure 2A), where the non-challenged 0.1% YGT birds had 2-fold higher expression compared to the other three treatments. There were no interactions on d 10 in the thymus or bursa or on d 14 in any of the immune organs observed.

The main effect of challenge could be seen on d 10 in the bursa, with significantly decreased expression in challenged birds compared to non-challenged birds (*p* = 0.0114; Figure 2C).

### 3.3. IL-17F mRNA Expression Levels (Table 2; Figure 3)

**Figure 3:**
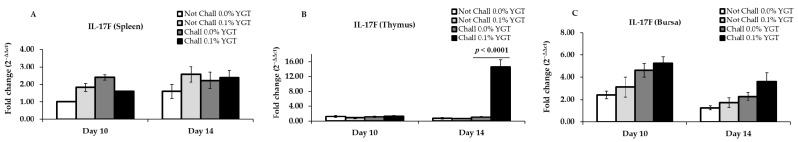
Effect of 2-way interaction (dietary β-glucan × *Eimeria* challenge) on abundance of IL-17F mRNA in the spleen (**A**), thymus (**B**), and bursa (**C**) of broiler chicks at d 10 and d 14. Relative gene expression (2^−∆∆Ct^) was calculated using the ∆∆Ct method with glyceraldehyde-3-phosphate dehydrogenase (GAPDH) as the endogenous control and the average ∆Ct value of unchallenged, 0.0% YGT control group at d 10 and 14 for each immune organ as the calibrator. Data are presented as least squares means + SEM. Not Chall = not challenged; Chall = challenged with *Eimeria*; YGT = Auxoferm YGT, *Saccharomyces cerevisiae*-derived β-glucan.

The dietary β-glucan treatment and *Eimeria* challenge resulted in a significant interaction on IL-17F mRNA levels in the spleen on d 10 and in the thymus on d 14. In the spleen on d 10, three treatments had higher expression relative to the non-challenged 0% YGT treatment, and the challenged 0% YGT had the highest fold change increase compared to the control (*p* < 0.0001; Figure 3A). On d 14, the challenged birds supplemented with 0.1% YGT had a 12-fold increase in thymus IL-17F mRNA expression compared to control (*p* < 0.0001; Figure 3B). There were no significant interactions on d 10 in the thymus or bursa, or on d 14 in the spleen or bursa.

The main effect of an *Eimeria* challenge appeared to increase IL-17F expression in the bursa on both d 10 and 14 (*p* = 0.0114 and *p* = 0.0048; Figure 3C) compared to non-challenged birds.

### 3.4. IFN-γ mRNA Expression Levels (Table 2; Figure 4)

**Figure 4:**
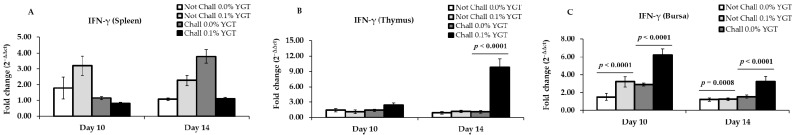
Effect of 2-way interaction (dietary β-glucan × *Eimeria* challenge) on abundance of IFN-γ mRNA in the spleen (**A**), thymus (**B**), and bursa (**C**) of broiler chicks at d 10 and d 14. Relative gene expression (2^−∆∆Ct^) was calculated using the ∆∆Ct method with glyceraldehyde-3-phosphate dehydrogenase (GAPDH) as the endogenous control and the average ∆Ct value of unchallenged, 0.0% YGT control group at d 10 and 14 for each immune organ as the calibrator. Data are presented as least squares means + SEM. Not Chall = not challenged; Chall = challenged with *Eimeria*; YGT = Auxoferm YGT, *Saccharomyces cerevisiae*-derived β-glucan.

Although there was no interaction between diet and challenge on any of tissues at d 10, there were significant interactions on d 14. Interestingly, on d 14, expression in the spleen of non-supplemented challenged birds was 3-fold higher compared to the non-challenged control (*p* = 0.0011), but the challenged birds supplemented with 0.1% YGT had similar mRNA expression to the non-challenged control (Figure 4A). On d 14 in the thymus, challenged birds supplemented with 0.1% YGT had a 9-fold increase in mRNA expression compared to the other three treatments (*p* < 0.0001; Figure 4B). There was also a significant interaction in the bursa on d 14, with YGT supplementation to challenged birds increasing mRNA expression by about 2-fold (*p* = 0.0152; Figure 4C).

No two-way interactions were observed on d 10, but there were main effects of either diet or challenge on mRNA expression. In the bursa, YGT supplementation increased d 10 IFN-γ expression (*p* < 0.0001; Figure 4C). Challenged birds had decreased expression in the spleen, but increased expression in the bursa on d 10 (*p* = 0.0033 and *p* < 0.0001; Figure 4A,C).

### 3.5. iNOS mRNA Expression Levels (Table 2; Figure 5)

**Figure 5:**
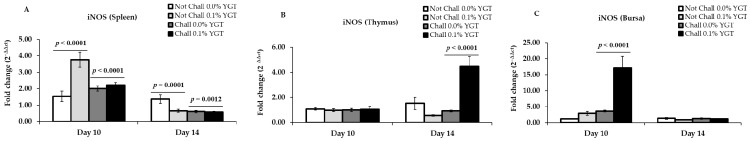
Effect of 2-way interaction (dietary β-glucan × *Eimeria* challenge) on abundance of iNOS mRNA in the spleen (**A**), thymus (**B**), and bursa (**C**) of broiler chicks at d 10 and d 14. Relative gene expression (2^−∆∆Ct^) was calculated using the ∆∆Ct method with glyceraldehyde-3-phosphate dehydrogenase (GAPDH) as the endogenous control and the average ∆Ct value of unchallenged, 0.0% Y1GT control group at d 10 and 14 for each immune organ as the calibrator. Data are presented as least squares means + SEM. Not Chall = not challenged; Chall = challenged with *Eimeria*; YGT = Auxoferm YGT, *Saccharomyces cerevisiae*-derived β-glucan.

In the spleen and bursa on d 10, there was a significant interaction between diet and challenge. In the spleen, non-challenged birds supplemented with 0.1% YGT had a 3-fold increase in iNOS mRNA expression (*p* = 0.0040), but both the challenged treatments were similar to the non-challenge control birds (Figure 5A). In contrast to the spleen, d 10 bursal iNOS expression was 15-fold higher in challenged birds supplemented with 0.1% YGT (*p* = 0.0095; Figure 5C). On d 14, there was a significant interaction in the thymus, where challenged birds receiving YGT supplementation had a 3-fold increase in expression compared to the non-challenged control, and both the non-challenged 0.1% YGT supplemented and challenged non-supplemented birds had decreased expression relative to the non-challenged control (*p* < 0.0001; Figure 5B).

On d 14, there was a main effect of diet on the spleen, where supplementation decreased iNOS mRNA expression compared to non-supplemented treatments (*p* = 0.0466; Figure 5A). There was also a main effect of challenge on the spleen at d 14, in which expression was decreased compared to non-challenged birds (*p* = 0.0266; Figure 5A).

### 3.6. MIF mRNA Expression Levels (Table 2; Figure 6)

**Figure 6:**
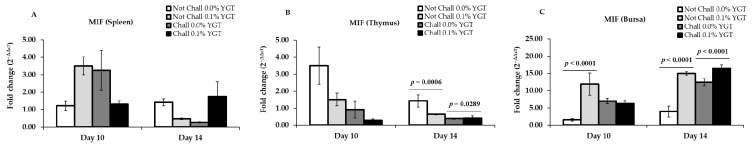
Effect of 2-way interaction (dietary β-glucan × *Eimeria* challenge) on abundance of MIF mRNA in the spleen (**A**), thymus (**B**), and bursa (**C**) of broiler chicks at d 10 and d 14. Relative gene expression (2^−∆∆Ct^) was calculated using the ∆∆Ct method with glyceraldehyde-3-phosphate dehydrogenase (GAPDH) as the endogenous control and the average ∆Ct value of unchallenged, 0.0% YGT control group at d 10 and 14 for each immune organ as the calibrator. Data are presented as least squares means + SEM. Not Chall = not challenged; Chall = challenged with *Eimeria*; YGT = Auxoferm YGT, *Saccharomyces cerevisiae*-derived β-glucan.

The dietary treatment and *Eimeria* challenge resulted in a significant interaction on MIF mRNA expression levels on both d 10 and d 14. On d 10 in the spleen, YGT supplementation in the challenged birds decreased expression by 3-fold compared to the non-challenged 0.1% YGT supplemented treatment and the challenged non-supplemented treatment (*p* = 0.0058; Figure 6A). In contrast, by d 14, MIF expression in the spleen was higher in the challenged birds supplemented with YGT, while the non-challenged 0.1% YGT supplemented treatment and the challenged non-supplemented treatment had decreased expression levels relative to the non-challenged control and challenged 0.1% YGT supplemented treatments (*p* = 0.0100; Figure 6A). In the thymus on d 14, there was a significant interaction in which expression in three treatments was downregulated in relation to the non-challenged control birds (*p* = 0.0274; Figure 6B). On d 10 and 14 in the bursa, there were significant diet × challenge interactions on expression levels. On both days, three treatments had increased expression levels relative to the control. Specifically, on d 10, the non-challenged 0.1% YGT supplemented treatment had a 12-fold increase in expression compared to control, and a 5-fold increase compared to both challenged treatments (*p* < 0.0001; Figure 6C). On d 14 in the bursa, both the non-challenged birds and the challenged birds receiving YGT supplementation, as well as the challenged birds receiving no supplementation, had higher fold change relative to the control (*p* = 0.0022; Figure 6C).

The main effect of an *Eimeria* challenge significantly decreased MIF expression in the thymus on d 10 (*p* = 0.0139; Figure 6B).

## 4. Discussion

Yeast-derived β-glucans (1,3/1,6-β-d-glucans) can activate the innate, as well as the adaptive immune responses of mammals, yet their influence on avian immune systems still is not fully clear. This study explored the effect of dietary β-glucan supplementation on mRNA abundance of immune response genes in major immune organs of broiler chicks with or without an *Eimeria* species challenge. The results often correlated in a two-way interaction between the two factors (dietary β-glucan supplementation, 0.0 or 0.1% YGT; no challenge or *Eimeria* challenge) on mRNA abundance of IL-10, IL-18, Il-17F, IFN-γ, iNOS, and MIF in spleen, thymus, and bursal tissues. IL-10 is an important regulatory cytokine for balancing host responses to pathogens and is secreted by antigen presenting cells (APCs) such as macrophages and dendritic cells. The ability of *Eimeria* to upregulate IL-10 negatively regulates immune responses by suppressing antigen presentation in broilers fed dried algae β-1,3-glucan [12]. Also, IL-10 can prevent further inflammation by suppressing IFN-γ production, which is a pro-inflammatory cytokine [29,30]. Expression of IL-10 mRNA at d 10 was upregulated in spleen and bursal tissues due to β-glucan × challenge interaction, as well as in the thymus on d 14. Moreover, IL-10 mRNA expression at d 14 was upregulated in spleen, thymus, and bursal tissues in the challenged and non-challenged birds. Production of IL-10 increased following an *Eimeria* challenge in broiler chicks at d 14 [5,30,31,32]. As a key immunoregulatory cytokine, IL-10 can potentially be used as a biomarker in chickens for *Eimeria* infection or to improve vaccine efficacy [33]. Challenging chickens with *E. tenella* revealed a significant positive correlation between lesion scores in the ceca and IL-10 [34]. This IL-10 response to an *Eimeria* challenge may be dependent on levels of other genes in the intestine, including TGF-β, IFN-γ, and some in the TNF family, or even in the spleen and thymus [35], in addition to the ceca, duodenum, and jejunum [36]. Supplementation of whole yeast cell product (1,3/1,6-β-glucans) in layer pullets and hens regulated the expression of IL-10 mRNA in the cecal tonsils post-coccidial challenge [11].

Interleukin-18 is another pro-inflammatory cytokine produced mainly by macrophages. IL-18 works in concert with other cytokines (e.g., IL-12) to induce a cell-mediated immune response to a pathogen. It mainly targets Th1 cells, which secrete IFN-γ that in turn can activate macrophages [37]. Expression of IL-18 mRNA at d 10 was slightly downregulated in the spleen due to β-glucan × challenge interaction, while no significant differences due to interactions of diet × challenge were observed in the thymus or bursa at d 10 and d 14. Further, IL-18 expression was greater in the ileum of birds receiving a β-glucan (yeast cell wall) supplemented diet, which might be associated with increased expression of Toll-like receptors and macrophage mannose receptor [25]. The downregulation of IL-18 observed in this study suggests an anti-inflammatory influence of β-glucan. Similar results have been noted in mammalian species in which levels of pro-inflammatory cytokines (e.g., IL-1β, IL-6, and TNF-α) were diminished due to β-glucan treatment [38,39]. Also, the production of IL-10 was augmented, while feeding β-glucan [38]. However, these cited reports examined the β-glucan effects during different challenge conditions in non-avian species. These varying results may be attributed to the variety of sources and derivation protocols of β-glucans, different tissue samples and application methods, as well as the challenge model employed.

Interleukin-17F is a pro-inflammatory cytokine that functions in gut homeostasis [40]. Much like IL-17A, IL-17F is secreted by various leukocyte populations such CD4+ and CD8+ T cells, NK cells, and neutrophils [41,42]. There were significant interaction effects on IL-17F in the immune organs. On d 14, IL-17F mRNA expression in the thymus and bursa was greater in the challenged birds when supplemented with β-glucan, which was also elevated at d 10 in the bursa, suggesting that dietary β-glucan acted as an anti-inflammatory agent. Abundance of IL-17F mRNA differed among tissues and was produced by a wider array of cell types and tissues and is involved in more biological activities than is IL-17A [43,44]. A previous study [40] reported that transcripts of IL-17F in *Eimeria*-infected chickens were abundantly found in all intestinal tissues as compared to the spleen, thymus and bursa. Moreover, IL-17F expression was increased in the thymus and bursa more than the spleen. Further, intestinal tissues of chickens infected with *E. tenella* and *E. maxima* had greater levels of IL-17F RNA levels than those from non-infected controls [40].

Interferon-γ is a key cytokine that functions in regulating innate and adaptive immune responses. It promotes Th1 cell differentiation and enhances activation and function of APCs [45]. Expression of IFN-γ mRNA was upregulated in the spleen, thymus, and bursa due to β-glucan × challenge interactions at d 14. In the bursa, dietary β-glucan supplementation significantly influenced IFN-γ expression dynamics as mRNA abundance of IFN-γ was upregulated in the non-challenged and challenged birds on d 10 and d 14. Zhang et al. (2008) also reported higher levels of this cytokine in broilers on diets with added β-glucans. Conversely, another study reported that abundance of IFN-γ mRNA was downregulated in 7-d-old chicks fed a β-glucan supplemented diet [18]. For coccidiosis, upregulation of IFN-γ and relative downregulation of IL-10 appear to be features of birds selected for disease resistance [46]. This variability in the immunomodulatory effects of β-glucans can also be due to their physical properties and their extraction methods [47].

As presented earlier, there is a myriad of cytokines that can direct immune responses, both innate and adaptive. In response to antigens or certain chemotactic agents, macrophages can upregulate the production of the iNOS enzyme, which promotes nitric oxide production. As a result of its reaction with superoxide anions, nitric oxide enables the generation of toxic derivatives that subsequently allows macrophages to proficiently kill invading pathogens [45]. The abundance of iNOS mRNA was influenced by the interaction of β-glucan and *Eimeria* challenge in the spleen and bursa at d 10 and in the thymus at d 14. In the spleen and bursa, expression of iNOS was upregulated in the challenged birds at d 10 and downregulation was observed at d 14 due to 0.1% YGT treatment and *Eimeria* challenge. Levels of iNOS were amplified due to the challenge and β-glucan supplementation compared to the control groups. These findings lead us to suggest that β-glucans exert an anti-inflammatory function in the absence of a challenge; however, during an intracellular infection, in this case *Eimeria*, β-glucans can enhance innate responses to the pathogen. Similar findings were reported in mammalian studies where the expression of iNOS and the resulting production of nitric oxide were observed in animals under β-glucan treatment [48,49]. Because of the nonspecific immune response, nitric oxide can exert an effective role in host defense mechanisms against invading microbes [48].

Macrophage migration inhibitory factor (MIF) is a pleiotropic cytokine produced by macrophages, dendritic cells, and T-cells, and influences the production of several inflammatory molecules [50]. It has been reported that high expression levels of MIF could result in anti-inflammatory events [51,52]. While considered essential in adaptive immune responses, MIF also plays a role in innate immunity [53]. On d 10 and d 14, mRNA abundance of MIF was influenced by β-glucan × challenge interaction. However, mRNA expression of MIF was upregulated in the bursa of non-challenged and challenged birds fed 0.1% β-glucan and downregulated in the thymus compared with control-fed birds at d 10 and d 14. Similarly, another study reported that the expression levels of MIF and other cytokines in intraepithelial lymphocytes mediated host defenses in *E. maxima*-challenged chickens [54]. MIF participates in regulating the expression of key molecules in the early immune response to different protozoa infections [50]. It has been observed that broilers fed dried algae β-1,3-glucan during an *Eimeria* challenge exhibited an increase in the number of total APCs (immune cells expressing MHC-II), including macrophages. Such an outcome suggests that recruitment of APCs to the small intestine of broilers during coccidiosis can be due to supplemental dried algae in their diets [12].

## 5. Conclusions

In conclusion, the reported findings show differential time effects on several gene targets in immune tissues in response to the dietary β-glucan, *Eimeria* challenge, and their interactions. Manifested in the thymus at d 14 were significant differences in mRNA abundance of IL-10, IL-17F, IFN-γ, iNOS, and MIF. However, in the thymus at d 10, there were no differences in IL-10, IL-18, IL-17F, IFN-γ, and iNOS mRNA expression due to the treatments. As previously shown [4], dietary β-glucan supplementation to *Eimeria*-challenged birds afforded a protective effect against coccidiosis infection in broiler chickens, which can be partly correlated with the observed later response that better corresponds with time of assessing pathology (lesion scores). As such, further more comprehensive studies could focus on additional measurements of immune response to better elucidate the timing of cytokine production and correlate that with immune protection. Additionally, one could assess the potential protective effects of other branched and linear β-glucan products extracted from other sources to better understand the modulation of immune response genes during a coccidiosis challenge, and perhaps other enteric stressors, to further enrich the knowledge of such products and their application in the field.

## Figures and Tables

**Table 1:** Primer pairs used for quantitative real-time PCR ^1^.

Target	Accession No.	Nucleotide Sequence (5′ → 3′)
GAPDH_F	NM_204305	CCTAGGATACACAGAGGACCAGGTT
GAPDH_R		GGTGGAGGAATGGCTGTCA
IL-10_F	NM_001004414	CGCTGTCACCGCTTCTTCA
IL-10_R		CGTCTCCTTGATCTGCTTGATG
IL-18_F	NM_204608	AGGTGAAATCTGGCAGTGGAAT
IL-18_R		TGAAGGCGCGGTGGTTT
IL-17F_F	XM_426223	CGCTTCCCCCAGGTGATT
IL-17F_R		CGCTTCCCCCAGGTGATT
IFN-γ_F	NM_205149	GCTCCCGATGAACGACTTGA
IFN-γ_R		TGTAAGATGCTGAAGAGTTCATTCG
iNOS_F	D85422	CCTGTACTGAAGGTGGCTATTGG
iNOS_R		AGGCCTGTGAGAGTGTGCAA
MIF_F	M95776	GCCCGCGCAGTACATAGC
MIF_R		CCCCCGAAGGACATCATCT

^1^ Primers designed by the Primer Express software (Applied Biosystems, Foster City, CA, USA). Abbreviations: F = forward, R = reverse; GAPDH = glyceraldehyde-3-phosphate dehydrogenase; IL = interleukin; IFN = interferon; iNOS = inducible nitric oxide synthase; MIF = macrophage migration inhibitory factor.

## Data Availability

Not applicable.
